# The importance of collegial support and a caring workplace culture for newly qualified nurses in becoming confident during their transition: a multi method study

**DOI:** 10.1186/s12912-025-04137-y

**Published:** 2025-11-22

**Authors:** Anette Tast, Jonas Vaag, Karin Bölenius, Anne Kasén, Yvonne Hilli

**Affiliations:** 1 Faculty of Nursing and Health Sciences, Nord University, 1490, Bodø, 8049 Norway; 2 Faculty of Social Sciences, Nord University, 1490, Bodø, 8049 Norway; 3 Present Address: Department of Psychology, University of Inland, P.O. Box 400, Elverum, 2418 Norway; 4 Department of Nursing, Faculty of Medicine, Umeå University, Umeå, 901 87 Sweden

**Keywords:** Caring science, Collegial support, Hermeneutics, Multi method, Newly qualified nurse, Transition, Workplace culture

## Abstract

**Background:**

Newly qualified nurses (NQNs) often face stress and lack confidence when transitioning to professional practice. Addressing these challenges is crucial for their development and overall well-being. The way NQNs are welcomed and supported in the workplace is a critical factor influencing their transition experience as they learn to be nurses in the new workplace.

**Aim:**

To deepen the understanding of the meanings of collegial support and workplace culture, and how these conditions promote mentees’ process of becoming confident nurses during their transition to professional practice.

**Method:**

An explanatory sequential design with a multi method (QUAL-quan) triangulation approach guided by caring science and Gadamer’s philosophical hermeneutics was employed. Pre- and post-tests were used to evaluate psychosocial conditions in the workplace over time among NQNs (*n* = 27). Following the intervention, NQNs (*n* = 19) participated in focus group interviews. Triangulation served as a methodological metaphor to integrate qualitative and quantitative data.

**Findings:**

The qualitative and quantitative findings point to the importance of a respectful, supportive, and positive workplace culture in enhancing the meaning of work, fostering a sense of fellowship, and building confidence and self-efficacy among NQNs. The results of the descriptive study demonstrate that social support and a sense of fellowship are highly valued, with no changes observed over time between the pre- and post-tests. Qualitative data support this, emphasising the significance of a caring workplace culture and collegial support.

**Conclusion:**

The findings of this study demonstrate the considerable importance of developing a caring workplace culture that welcomes and respects NQNs as new colleagues. Value-conscious leadership is crucial in fostering such a culture by setting the tone in the workplace, thereby creating conditions that support continuous learning, fellowship, and overall well-being. This study reinforces the importance of supportive colleagues and a compassionate workplace culture in boosting NQNs’ confidence and their development into confident nurses.

**Clinical trial number:**

Not applicable.

**Supplementary Information:**

The online version contains supplementary material available at 10.1186/s12912-025-04137-y.

## Background

Newly qualified nurses (NQNs) face challenges when transitioning to professional practice and often experience stress and a lack of confidence [1, 2]. Recognising and addressing the challenges NQNs encounter during this pivotal period in their personal and professional development, as well as their continued commitment to the profession and overall well-being, is essential. How the NQNs are welcomed and supported in the workplace has been identified as a critical factor influencing their transition experience as they learn to be nurses in the new workplace [3–5]. While NQNs experience an overwhelming sense of responsibility and a lack of confidence in their early practice [6], further research is needed to explore the meaning of collegial support and workplace culture for a smooth transition.

This study is part of a larger Nordic action research project between two universities, hospitals and municipalities in northern Norway and northern Sweden. A mentorship programme was developed in close collaboration with registered nurses and leaders from various hospitals and municipalities. The mentorship programme was tested over eight months in various intervention units across hospitals and five municipalities. This study is a post-intervention evaluation aimed at exploring the meanings of collegial support and workplace culture from the perspective of the NQNs, the mentees’ process of becoming confident nurses.

## Literature review

Several studies report that NQNs experience significant responsibilities and stress during the transition, negatively impacting their development, well-being, and professional commitment. However, many find the profession satisfying and meaningful, viewing this period as a time of personal growth [1, 5, 7]. The shift from being a student to becoming a registered nurse is a complex process, influenced by various personal, relational and organisational factors [3]. Although NQNs may encounter similar challenges, their support needs are individual, unique, and context-specific [8]. The NQNs’ personality and values, especially concerning confidence-building, are vital for a smooth transition process [9]. The commonly reported barriers and stressors include high workload, inadequate staffing, and demands to manage multiple roles. Additional stressors include unsupportive workplace culture and incivility [5, 9, 10]. Negative experiences, such as feeling overwhelmed, disrespected, stressed, isolated, lacking confidence and inadequately prepared, can lead to ill-being [2, 5, 11]. Moderately high levels of burnout are reported among NQNs, particularly concerning emotional exhaustion during their first year of employment [1]. The adverse effects of overwhelming or prolonged stress can manifest early in a nurse’s career. Therefore, it is crucial to introduce preventive measures early on to avoid far-reaching consequences [12].

Transition programmes have been designed to facilitate the transition from student to professional nurse. They have been demonstrated to contribute to increased confidence, enhanced job satisfaction and dedication to the profession [8, 13, 14]. The duration of support in these programmes and the content can vary from a few weeks to two years [14–16]. The sense of being supported by mentors, colleagues, and leaders has been reported to impact their transformation significantly [7, 8]. A systematic review concluded that most programmes emphasise technical skills, with increased confidence being the most commonly reported outcome [15]. Still, it is difficult to determine whether this is due to the programmes or whether it is the time that contributes [3, 15]. According to current evidence, the impact of programmes is inconclusive regarding competence, confidence and attrition rates [14, 16]. Nevertheless, a supportive workplace has been identified as an effective and meaningful approach. Successful transitions are facilitated by progressive transition programmes and positive and supportive workplaces integrated into everyday work [10, 17]. Characteristics of a supportive workplace have been reported to include being accepted by colleagues and the team, efficient communication, sharing experiences and knowledge with colleagues, the availability and accessibility of informal support and providing opportunities for learning and constructive feedback [14]. Positive experiences such as feeling joy and safety, belonging to a team, feeling valued, and learning from and being supported by colleagues, help build confidence in one’s abilities. Collegial support has been shown to enhance confidence and self-efficacy [10, 18]. This enhances opportunities for personal and professional growth, well-being, and commitment to the profession [4, 5, 8, 9, 19].

Leadership support is crucial in cultivating a culture of trust by organising conditions for development, offering opportunities for varied clinical experiences, and facilitating a supportive learning environment with access to formal and informal support. NQNs request an ongoing dialogue with their leaders regarding development issues and any questions or problems that may arise during the transition period. Failure to fulfil promises of support can lead to disappointment and feelings of being abandoned [13, 19, 20].

The psychosocial conditions within the workplace culture appear to significantly influence the transition experiences of NQNs [3, 4], which include supportive colleagues, essential for NQNs to feel secure while learning and developing as nurses in their new roles [7, 8, 13, 18]. An earlier study concluded that supportive workplace cultures can offset inadequate mentorship during this transition [1]. There is a deficiency of research investigating the significance of the workplace culture in supporting NQNs during the transition. A review revealed a notable gap in interventions addressing the psychosocial implications of transition and their connection to professional identity and well-being [15]. This study addresses this gap from a caring science perspective by investigating the meanings of collegial support and a nurturing workplace culture to support NQNs in becoming confident nurses during the transition.

## Theoretical perspective

This study’s theoretical perspective is rooted in caring science, encompassing an ethos of love, compassion, and respect for human dignity. The human being is seen as an entity of body, soul, and spirit, possessing unique desires and needs. There is a belief in the human capacity to constantly learn and evolve from actual to potential and striving for balance and development [21]. While individuals are exceptional, they yearn to belong to a fellowship. Fundamental values, ethos, reside deep within the human being, serving as a driving force that motivates and guides an individual’s actions and will become evident in their way of being, and in the physical space where individuals meet and interact [22]. Staying connected to one’s innermost self is essential for an individual’s experience of meaning in life, health and well-being. When individuals can act following their values, they feel respected and experience a sense of belonging, which enhances their self-esteem and well-being [22].

According to Gadamer [23] people learn and are moulded by their environment and culture through fellowship with others. Learning is considered more than merely acquiring skills, behaviours and attitudes. It is an ongoing internal process of becoming, a formation (in German, “Bildung”). A process where internal appropriation and application are essential for developing a new understanding. When values, knowledge and actions are intertwined with people’s pre-understanding, a fusion of horizons occurs [23, 24]. This process of ‘becoming’ arises from an individual’s personal development and continues throughout life [23]. Thus, professional ways of becoming a nurse develop gradually as knowledge, experience and action are integrated over time [25].

## Methods

### Aim

This study aimed to deepen the understanding of the meanings of collegial support and workplace culture, and how these conditions promote mentees’ process of becoming confident nurses during their transition to professional practice.

The research question was: What support from colleagues and workplace culture facilitates mentees’ becoming confident nurses during their transition?

### Research design

This study employed an explanatory sequential design with a multimethod (QUAL-quan) triangulation, privileging an approach guided by caring science and Gadamer’s philosophical hermeneutics [21, 23, 26]. The epistemology and methodology form the core of the study, guided by the qualitative tradition, recognising the importance of listening to the subjective meanings individuals construct in a specific social context. The quantitative follow-up, descriptive survey plays a second role, assisting in the elaboration and interpretation of the overall, qualitatively driven core research question(s). This guide the process of obtaining a deeper and more nuanced understanding of how NQNs experienced support from colleagues and the workplace culture. Triangulation was employed as a metaphor to integrate qualitative and quantitative data, examining how NQNs perceived the support they received during their transition [26–29]. The study was reported following the Consolidated criteria for reporting qualitative research (COREQ) checklist [30] and the Good reporting of a mixed methods study (GRAMMS) checklist [31].

### The intervention and setting

**Fig. 1:**
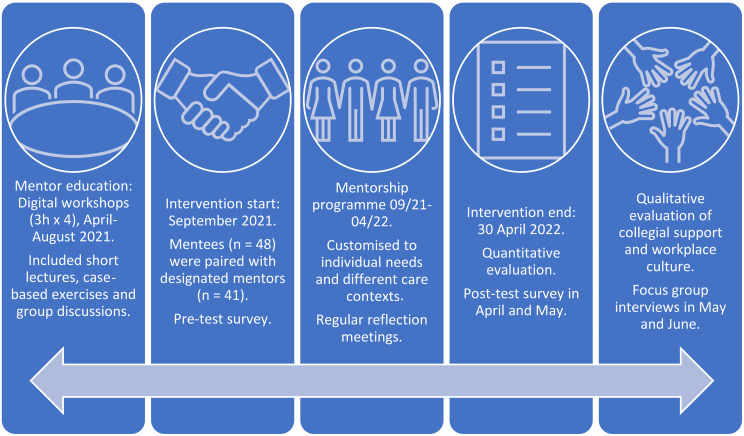
Overview of the intervention, the timeline and its contents

In this project, a mentorship programme was developed from April to August 2021, in close collaboration with registered nurses and leaders from various participating hospitals and municipalities in Norway and Sweden. The mentorship programme included regular reflection meetings, which were held more frequently at the beginning and gradually less often as time went on. The programme was customised to meet the mentees’ individual needs and the capacity of each care context. Mentors received education through four digital workshops (3 h each), covering the following themes:mentorship and the workplace culture, leadership responsibility, and reflective conversations. An overview of the intervention, the timeline and its contents is shown in Fig. 1. The mentorship programme was tested from September 2021 to April 2022 in diverse intervention units in two hospitals and five municipalities.

### Participants

All mentees (*n* = 48) who participated in the intervention were invited to the evaluation process. The inclusion criteria were as follows: NQN mentee, with work experience ranging from one to 18 months and employed at least 75%, assigned to one or more mentors, and able to communicate in Norwegian or Swedish. At the commencement of the intervention, the participants had between three and 18 months of work experience. They worked in various specialised healthcare settings, including medical, surgical, and emergency units. In municipal healthcare, they worked in nursing homes, rehabilitation units, or in-home care. Table 1 presents an overview of the participants’ demographics in the pre- and post-test and the focus group interviews.

**Table 1:** Overview of participants’ demographics in pre-and post-test (*n* = 27) and focus group interviews (*n* = 19)

Demographics of participants	Pre- and post-test (*n* = 27)	Focus group interview (*n* = 19)
**Gender**		
Women Men	23 (85%) 4 (15%)	16 (84%) 3 (16%)
**Age**		
≥ 25 years 26–30 years ≤ 31 years	18 (67%) 6 (22%) 3 (11%)	10 (53%) 5 (26%) 4 (21%)
**Work experience at the start of the intervention**		
1–3 months 4–6 months 7–18 months	5 (19%) 16 (59%) 6 (22%)	3 (16%) 12 (63%) 4 (21%)
**Workplace**		
**Specialist health care**		
Medical, surgical, and emergency units.	15 (56%)	8 (42%)
**Municipal health care**		
Home care, rehabilitation units, and nursing homes.	12 (44%)	11 (58%)
**Employment**		
100% 75%	23 (85%) 4 (15%)	17 (89%) 2 (11%)

### Data collection

#### The pre- and post-test study

All participating (*n* = 48) mentees (NQN) were asked to fill out an online self-administered survey at two points: the pre-test (baseline) before the start of the intervention in September 2021 and the post-test immediately after the end of the intervention in April-May 2022. Two response reminders were sent. Participants who completed the pre- and post-tests (*n* = 27) were included to identify potential changes over time.

#### The questionnaire

The pre- and post-tests used selected parts of the Copenhagen Psychosocial Questionnaire (COPSOQ III), an internationally validated tool for evaluating psychosocial conditions in the workplace [32]. The questionnaire has been validated in the Scandinavian context and welfare in Norwegian and Swedish healthcare settings [33, 34]. It comprised background questions and questions covering self-reported assessments of psychosocial conditions in the workplace, including the meaning of work, social support, sense of community, stress, sleeping troubles, burnout and self-efficacy. All items included the core variables, and participants indicated their level of agreement with the statements on a Likert-type scale.

*The meaning of work* was assessed using a 5-point scale, ranging from “to a very large extent” (5), to a large extent (4), somewhat (3), to a small extent (2) to a very small extent (1)”. The response options for *social support from leaders* and *colleagues*, and the *sense of community at work*, ranged from “always (5), often (4), sometimes (3), seldom (2), to never/hardly ever (1)”. To evaluate well-being, items on *sleeping troubles, burnout*, and *stress* were used, with response options ranging from “all the time (5), a large part of the time (4), part of the time (3), a small part of the time to (2) not at all (1)”. *Self-efficacy* was evaluated on a 4-point scale in Norway, with the response options from “fits perfectly (4), fits quite well (3), fits a little bit (2), to does not fit” (1)” [34]. In Sweden, occupational self-efficacy (OSE) [35] was measured on a 6-point scale with response options ranging from “not at all true (1) to completely true (6)”. High values indicate a strong sense of OSE.

### The focus group interviews

Focus group interviews were conducted to gain a more nuanced and in-depth understanding of the meaning of collegial support and workplace culture during their transition [36]. Recruitment of mentees for focus group interviews began immediately after the intervention concluded on April 22. All intervention participants (*n* = 48) were invited to participate via email and telephone. Five mentees had changed workplaces, and no contact information was available. Nine declined to participate, nine did not respond to the invitation, and six were unable to join due to workload or other reasons. A total of 19 participants took part, comprising 16 women and three men. Their ages ranged from 22 to 43 years, with a mean age of 25.

Digital focus group interviews were conducted through May and June 2022. Three interviews were conducted in Norway (n = 13) and two in Sweden (n = 6) during their working hours. Each focus group consisted of two to seven participants, including one experienced and one junior researcher, who conducted interviews in each country. A relaxed atmosphere was created to encourage conversation and dialogue, allowing participants to share personal experiences. The research team developed an interview guide (supplementary material) to evaluate the importance of support for NQNs within the workplace culture during the intervention. The same interview guide was used in both countries to ensure consistency across all interviews. The interviews commenced with open-ended questions, such as ‘Can you please tell us about the workplace culture?’, ‘Could you please tell us about the support you have received that has promoted your learning and development?’ and ‘Can you please tell us if you have experienced promoting or hindering conditions for your learning and development in the workplace?‘. Participants were encouraged to elaborate on their thoughts with follow-up questions, such as ‘Can you expand on what it means to you?’ The participants’ statements were confirmed by repeating and summarising shared understanding during the interviews. The interviews were conducted using Microsoft Teams™ or ZOOM™, and field notes were taken simultaneously. The interviews lasted between 69 and 113 min and were recorded and transcribed verbatim.

### Data analyses

#### The descriptive analyses of pre-and post-test

A descriptive analysis of the mentees’ self-rated perceptions of the psychosocial conditions in their workplaces was conducted using Statistical Package for the Social Sciences (SPSS) version 29. Mean scores and standard deviations for the whole group are analysed for pre- and post-tests. Due to the minor differences in the use of scales in measuring self-efficacy, this concept is presented separately for each country.

#### The interpretation of the focus group interview

The interpretation process, guided by Gadamer [23], involved the first author reading the data with an open, humble, and questioning attitude to capture the meaning of the entire text. The initial level of interpretation involved repeatedly reading the data while constantly asking questions to achieve a broader understanding, transitioning back and forth between the text as a whole and its various parts. During this phase, meaningful patterns gradually emerged. At the second level, the different parts and the whole text merged with pre-understanding, leading to a fusion of horizons and a new understanding occurred, known as the hermeneutic circle. The authors met numerous times, and once an agreement was reached, the final three themes were established and presented in Table 2. In the third level of interpretation, the authors contemplated the findings within the context of the theoretical perspective and prior studies. Through a process of fusion of horizons, a deeper and shared understanding was attained through dialogue and is presented in the discussion. The first author responsible for the data analysis engaged in a continuous reflective dialogue with the other authors throughout the process [23, 37].

**Table 2:** Examples of meaning units and themes

Meaning units	Themes
Being welcomed and feeling that you can ask questions of everyone. That people take the time to show you things you don’t know, that you have a good lunch together, that you laugh, that there is a good atmosphere. People are positive and supportive towards one another. This means a lot to me because I feel good, I enjoy being at work, and I want to learn more. That you make each other feel good, regardless of where you stand, whether you’re experienced or not, instead of blaming each other.	A workplace culture characterised by mutual respect and collegial fellowship
They [leaders] care about how you are doing at work. You feel that you are seen and cared for. The development dialogue was very good because you can discuss how things have been going and how you feel at work. If there has been anything difficult or if things are going well, you have time set aside to discuss it with your leader. It is doing that little bit extra [leader], even if you don’t always have time for it, it’s just the thought of having tried to create a pleasant moment. It does well.	Responsive leadership cultivating a caring culture
You learn a lot about yourself when you are with people in different life situations with different experiences, you learn to deal with situations that you didn’t think you could deal with yourself. It is stressful to feel that you’re constantly working against the wind, but at the same time, you get a fighting spirit. We are together, and there’s good cohesion between assistant nurses and nurses. It was forgotten that we were mentees, and we were thrown in at the deep end. We got a lot of support from leaders and more experienced nurses at the beginning, and then it disappeared.	Facilitating and hindering conditions in the workplace culture

### Triangulation

Triangulation was used as a methodological metaphor when integrating qualitative and quantitative findings, thereby deepening and nuancing the understanding of the meanings of collegial support and the workplace culture for NQNs during their transition. This approach connects the theoretical perspective to the empirical findings [29].

### Ethical considerations

Participants received written and oral information about the intervention, the study’s purpose, pre- and post-tests, and focus group interviews. All participants obtained informed consent before the intervention started, including for the surveys and the focus group interviews. Participation was voluntary, and they could withdraw their consent at any time. The researchers emphasised confidentiality throughout the whole process. All participants were pseudonymised and given a code, as this was a follow-up study. The key code was stored in a locked and secure place, following each country’s and organisation’s requirements. The study was conducted in accordance with the Declaration of Helsinki and approved by the Norwegian Agency for Shared Services in Education and Research (No.148896) and the Swedish Ethical Review Authority (Dnr 2020–06187). Researchers deleted all recordings directly after the transcription of the interviews. Data was anonymised during transcription and coded at the group level as NO.1–3 and SWE.1–2. After the final survey and analysis of the focus group interviews, the researchers deleted all the codes. Findings from the focus group interviews are presented on a group level to ensure participant anonymity.

## Findings

The findings are presented in three sections: the focus group interviews with the mentees, the outcomes from the longitudinal pre-and post-tests, and the integration of these empirical findings with the theoretical perspective.

### Qualitative findings

A pattern emerged from the rich data, emphasising the significance of a welcoming workplace culture. Mentees described an open and friendly workplace culture that provided support as a safe place for learning and development, fostering a spirit of fellowship and belonging. Leadership was pivotal in establishing these conditions by cultivating a nurturing culture that encouraged learning, development and overall well-being. Conversely, lacking support could result in stress and feelings of inadequacy, hindering professional growth. The findings are presented as a pattern of three complementary themes, without hierachy: *A workplace culture characterised by mutual respect and collegial fellowship, Responsive leadership cultivating a caring culture*, and *Facilitating and hindering conditions in the workplace culture.*

#### A workplace culture characterised by mutual respect and collegial fellowship

The mentees emphasised the importance of being invited and welcomed into the workplace and the team as new colleagues. They stressed the importance of being respected as a new colleague and feeling free to be unique with individual needs. Such a culture is characterised by a positive, open, and permissive atmosphere where mentees can learn from more experienced colleagues. This atmosphere encouraged mentees to dare to ask questions and was considered a safe and favourable arena for learning and development.

Being welcomed and feeling that you can ask questions from everyone, and that people take the time to show you things …that there’s a good atmosphere, that everyone is positive…That’s very important because I feel I can have a good time and want to be at work and learn more… (NO. 1)

Mentees wanted to contribute to cultivating a culture of mutual respect, support and recognition of each other’s resources through everyday gestures: “To see people and say: Hi…Ask if they needed help or be helpful when people come to ask for help” (NO.3). A supportive culture encouraged positivity, identified possibilities and fostered a friendly atmosphere. Such a culture focused on uplifting one another rather than on shortcomings: “that you try to do each other well, regardless of where you stand, whether you are experienced or not. Instead of trying to blame each other…” (NO. 2). A caring culture where the team had shared values and was driven towards common goals by recognising each other’s resources created a spirit of fellowship and sense of belonging which strengthened motivation, job satisfaction, and well-being.

…you learn something new every day, and even though you’ve had a hard time, you have good colleague support. We have high ceilings and joke with each other even though we can have serious moments. You encounter many new things and continually develop within your profession. No two days are the same. (SWE.1)

As the quote above describes, relationships between colleagues were considered crucial to maintaining a supportive and positive atmosphere, as well as strengthening the sense of belonging. Peer support from other mentees was important and often developed into friendships: “We’re friends in our spare time too… we’ve known each other since we started here” (NO.3). In workplaces with several mentees, they could form a peer support group, providing each other valuable support and sharing experiences.

It was good for us that we [mentees] got to know each other as a group, because since then we have reflected a lot as a team, as we are all new… I feel that it is vital for me… to have both new nurses and those who have been in the field for many years, who have a lot of different things to contribute. (NO. 2)

Regular and constructive feedback from more experienced colleagues helped mentees gain self-confidence, enabling them to recognise their progress and step beyond their comfort zones. The support fostered a feeling of belonging and teamwork: “…they cheer you on…look how much you have developed! They become like your team” (SWE. 2).

#### Responsive leadership cultivating a caring culture

The mentees emphasised the importance of having a responsive leader who promoted a caring culture in the unit, thus creating the conditions for professional development and progression. They appreciated having a relationship where expectations and responsibilities were clearly communicated, thus creating an environment conducive to their growth and ensuring the delivery of high-quality patient care.

You can tell them [leaders] how you’re feeling. They ask how you’re doing and if there’s anything they can do to make things easier… I feel that they care about how you’re doing at work, and that things are going how you intend… (NO. 2)

Feedback and confirmation from the leader were valuable, and the mentees requested regular developmental dialogues. Mentees who had such dialogues felt respected and that the leader cared for their development and well-being: “You feel that you are seen and cared for” (NO.2). During the dialogues, mentees could share their experiences as new nurses, difficulties at work and needs for further support. Moreover, the dialogue played an essential role in their development process and motivated them to focus on their career development: “I feel that the leaders could have had more understanding that you are new and don’t feel ready for certain things, just such a thing, and to have a conversation about it (SWE 2)”.

Mentees appreciated when the leaders showed their care by creating small moments of relaxation in the middle of a busy workday: “It’s doing that little bit extra [leader]… even if you don’t always have time for it, it’s just the thought of having tried to create a pleasant moment. It does well (NO. 1)”. This gesture fostered a positive atmosphere that enhanced the mentee’s sense of belonging, job satisfaction and well-being.

Some mentees indicated they lacked the leader’s support and expressed disappointment when promised support was not fulfilled. It could be about promised development dialogues that never took place or educational courses in which they were not allowed to participate.

#### Facilitating and hindering conditions in the workplace culture

The mentees valued a workplace culture with opportunities to develop personally and professionally. The daily interactions with patients and colleagues were rewarding and contributed to the sense of meaningfulness of the work. Building relationships with patients and making a difference in their lives were motivating. In addition, positive feedback from patients and relatives was encouraging.

…you learn a lot about yourself when you are with people in different situations in life with different experiences, you learn to deal with situations that you didn’t think you could deal with yourself… (SWE. 1)

A high workload and a fast work pace characterised the working days of several mentees. There was frequently a shortage of staff with the necessary skills, leading to increased responsibilities and extended working hours. All this contributed to enhanced stress and anxiety due to the lack of support in difficult situations. When work was well-organised and everyone understood their responsibilities, knowing what to do and where to find information, operations ran smoothly. Effectively managing the day required a strong team spirit and adaptation to the workplace culture, which cultivated a sense of fellowship: “… it’s stressful to feel that you’re constantly working against the wind, but at the same time, you get a fighting spirit… we are together, and there’s good cohesion between assistant nurses and nurses… (SWE.2)”. With the support of their colleagues, mentees learned to manage stress, multitask and prioritise effectively: “I’ve got to know how I cope with stress and it’s getting better. I’ve become better at handling lots of things at the same time. I hope it will improve over time (NO.1)”.

Mentees expressed that their need for ongoing support from leaders and colleagues to facilitate continuous learning and development sometimes was overlooked: “It was forgotten that we were mentees, and we were thrown in at the deep end… we got much support at the beginning and then it disappeared… (NO.2)”. Mentees perceived and pointed to the fact that they learned from being given responsibility, however, when they were given responsibilities and tasks, they did not feel ready for, it added the pressure: “…you learn a lot and that you can take such responsibility but also try to realise if this is my responsibility? …I feel that it puts more pressure on me… (SWE.1)”. Mentees who lacked support felt overwhelmed by their responsibilities and workload, expressing concern about their well-being and future careers as nurses. They experienced feelings of inadequacy and fatigue, negatively affecting their sleep and overall well-being:

How long can you stay in this game? I find it rather sad when you’re a mentee… It’s tiring to feel that you can’t be the nurse you wish to be because you lack the time for it. You don’t feel as though you’re fulfilling the reasons you entered the profession. You must constantly compromise with yourself to accomplish the most critical tasks, and it’s incredibly exhausting. (NO.2)

Working alone without support was stressful and could lead to feelings of inadequacy, abandonment and vulnerability. When some colleagues were unsupportive, especially in situations involving mistakes or shortcomings, it increased pressure and contributed to a sense of ill-being. In such situations, mentees expressed the need for a more constructive and helpful attitude from their colleagues.

... I wished for an experienced colleague to be there with me because I felt lonely and vulnerable… it doesn’t have to be a specific mentor or a colleague; it can be anyone involved in a similar situation. (SWE.1)

### Quantitative findings

Mentees’ experiences of psychosocial conditions were not changed over time between the pre- and post-tests. The meaning of work (M = 4.46–4.38), sense of community (M = 4.28–4.22), and social support from colleagues (M = 4.16–4.21) were rated most highly. Stress (M = 2.24–2.45), sleeping troubles (M = 2.33–2.14), and burnout (M = 2.81–2.99) were rated the lowest. In Norway, self-efficacy was rated (M = 2.88–2.93), and in Sweden, OSE was measured (M = 3.83–4.02). The descriptive statistics are presented in Table 3.

**Table 3:** Participants’ (*n* = 27) experiences of psychosocial conditions in the workplace

	Pre-test	Post-test
	Mean (SD)	Mean (SD)
Meaning of work^a^	**4.46 (0.57)**	**4.38 (0.59)**
Social support from supervisors^a^	3.70 (0.76)	3.71 (0.85)
Social support from colleagues^a^	**4.16 (0.63)**	**4.21 (0.60)**
Sense of community^a^	**4.28 (0.49)**	**4.22 (0.49)**
Sleeping troubles^b^	2.33 (0.86)	2.14 (0.81)
Burnout^b^	2.81 (0.93)	2.99 (1.03)
Stress^b^	2.24 (0.89)	2.45 (0.84)
Self-Efficacy^1, a^ Norway (*n* = 19) Sweden (*n* = 8)	2.88 (0.52) 3.83 (0.26)	2.93 (0.56) 4.02 (0.57)

^1^Self-Efficacy was measured with different scales for Norway (1–4 point) and Sweden (1–6 point). For items marked with^a^ a higher value is the ideal response. For items marked with^b^, a lower value is the ideal response

Note: The highest values are highlighted in boldface

### Triangulation of the findings

**Fig. 2:**
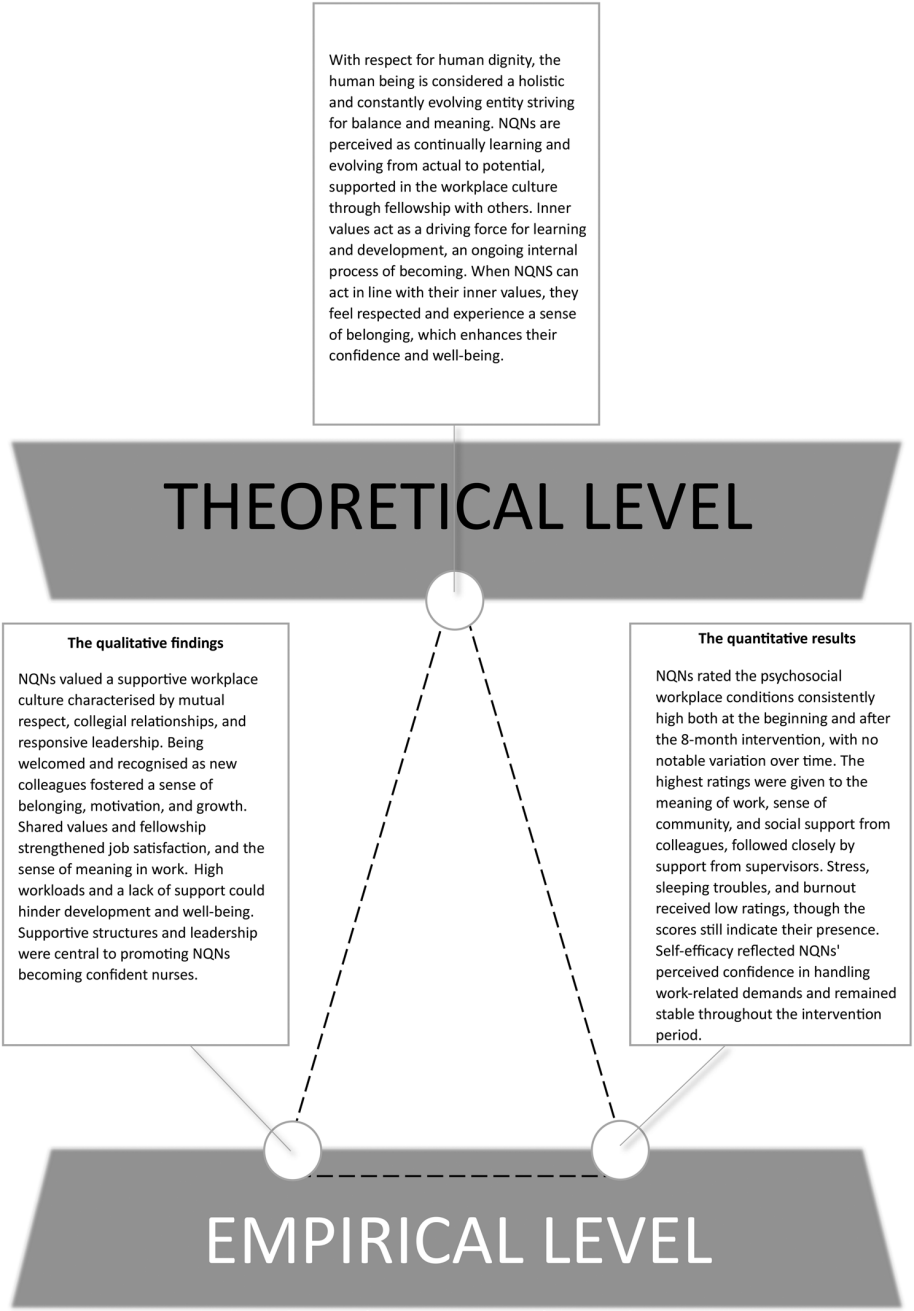
Triangulation of qualitative interview findings and quantitative pre-post survey data with the theoretical propositions

The integration of qualitative and quantitative empirical findings, alongside the theoretical propositions, is visualised through a triangle metaphor (see Fig. 2). The qualitative findings form the core of the synthesis, while the quantitative descriptive follow-up results offer a secondary layer that supports the interpretive process.

NQNs emphasised the importance of a respectful, supportive, and positive workplace culture. Being welcomed as new colleagues into a safe environment was seen as essential, enabling them to engage in learning and development at their own pace. A culture that promotes a spirit of fellowship enhances motivation, job satisfaction, and overall well-being, as stated in the theoretical propositions [21–23]. This is supported by the descriptive follow-up study, which shows that NQNs highly value social support from colleagues (M = 4.16–4.21) and the sense of community within the workplace culture (M = 4.28–4.22). Acting in accordance with shared values and common goals fostered a sense of belonging, which in turn strengthened their self-esteem and overall well-being, further supported by the theoretical propositions [22, 23].

NQNs found their work meaningful through opportunities for personal and professional development, rewarding interactions with patients and colleagues, and positive feedback. Building relationships and making a difference in patients’ lives were motivating factors. This is supported by high scores on the meaning of work in the descriptive results (M = 4.46–4.38) and further substantiated by the theoretical propositions underpinning the study [22, 23]. Support and fellowship among NQNs provided a safety net, enabling them to share experiences and learn from one another. Peer support and continuous learning over time reinforced their belief in their abilities, contributing to their overall self-efficacy. Regular and constructive feedback from more experienced colleagues helped NQNs recognise their progress and strengthen their self-confidence. This ongoing affirmation was regarded as particularly important in helping NQNs to feel capable of managing more complex clinical situations, as reflected in the quantitative ratings of confidence and self-efficacy (M = 2.88–2.93, and OSE M = 3.83–4.02).

NQNs valued responsive leadership that fostered a caring culture through regular development dialogues, which helped them recognise their progress and boost their self-efficacy. This was supported by the descriptive results, which showed that supervisor support was rated quite highly (M = 3.70–3.71), emphasising the importance of responsive leadership in fostering a caring workplace culture.

From the perspective of NQNs, the development of confidence was influenced by both facilitating and hindering conditions within the workplace culture. High workloads and staff shortages often resulted in increased responsibilities and stress; therfore, a supportive team was deemed essential in managing these challenges. A lack of support in such situations exacerbated stress and anxiety. Overwhelming responsibilities, coupled with feelings of inadequacy, negatively affected NQNs’ sleep and overall well-being. Although the quantitative results did not show particularly high levels, they still indicated the presence of sleeping troubles (M = 2.33–2.14), stress (M = 2.24–2.45) and burnout (M = 2.88–2.99), thus supporting the qualitative findings. Some NQNs expressed concerns about their well-being and future careers due to these factors.

According to NQNs, a supportive workplace culture can help reduce stress and enhance well-being. They rated various psychosocial aspects of their workplace culture highly throughout the intervention period, and the absence of temporal variation indicates a stable perception of collegial support and a caring workplace culture. This helped NQNs uphold their sense of meaning of work and maintain their overall well-being during the transition period, aligning with the theoretical propositions emphasising shared values, belonging, and the ongoing internal process of becoming [21].

## Discussion

This study aimed to deepen the understanding of the meanings of collegial support and the workplace culture in facilitating the development of NQNs into confident nurses during their transition to professional practice. The discussion highlights the findings and relates them to the theoretical perspective and previous research.

The findings in this study emphasised the importance of a supportive and nurturing workplace culture, where NQNs could engage, collaborate and learn alongside their peers and more experienced colleagues, as evidenced by previous studies [7, 9, 11]. In this study, NQNs appreciated a value-based culture characterised by mutual respect, commitment, responsibility and collegial support. Being welcomed and respected as new colleagues and included in the team, rather than being left outside, enhanced feelings of security and provided a nurturing space where NQNs could learn, develop, and progress at their own pace [7, 22]. In such a workplace culture, the core values—the ethos—were made visible through caring for one another, supporting the development, and promoting the well-being of NQNs, which led to feelings of belonging and trust [5, 21, 22]. When the prevailing tone of the culture was friendly and the spirit was helpful, a sense of fellowship was fostered [22]. Such collegial relationships created a home-like workplace where trust and friendship flourished, and they felt metaphorically at home [22, 38]. When NQNs were formed in a culture of care, characterised by mutual respect, true friendship and commitment, this was reflected in their relationships with peers and patients [22]. NQNs in this study emphasised the importance of being recognised, receiving constructive feedback, and learning with colleagues. Taking on more responsibility, along with available support, and sharing and reflecting on experiences with colleagues facilitated an internal appropriation of knowledge and its application in practice, thus developing a new understanding. Collegial support helped NQNs to broaden their understanding over time as they gained more experience. As values, knowledge and actions were intertwined and integrated over time, a fusion of horizons occurred, and the development continues as lifelong learning [23–25].

In this study, NQNs emphasised the importance of responsive leadership, which created a supportive culture and, thus, conditions for personal and professional development. This finding is consistent with a review [39], which underscores the vital role of leaders in actively supporting the development of NQNs. Caring and responsive leadership was demonstrated through genuine concern for their development by listening to and recognising their needs, providing feedback and conducting regular developmental dialogues. The responsibilities, at the appropriate level of expertise and expectations, were communicated by defining a clear role for the NQNs. As shown in a recent study [40], experienced nurses acting as mentors requested that leaders take responsibility for facilitating a clear organisational structure and providing support to ensure and promote a safe transition for NQNs. The findings in this current study are consistent with those in a recent meta-ethnographic study, where leaders and nurses perceived that a health-promoting work environment is grounded in core values, including respect, recognition, community and engagement. In such a workplace, leaders are aware of their power position and responsibilities, and they cultivate safe working conditions that encompass the work environment [41]. A value-conscious leadership and caring approach sets the tone for interaction and collaboration in the overall workplace culture, creating an atmosphere that promotes mutual respect, equality, and trust among nurses, as demonstrated in earlier studies [41–43]. A lack of promised leadership support could cause disappointment and harm NQNs’ confidence and development. This highlights the importance of leaders following through on their commitments and providing ongoing support during the transition. Earlier studies, as well as this study, suggest that the presence of responsive and value-conscious leadership can significantly impact NQNs’ job satisfaction and their intention to stay in the profession [13, 17, 43].

When the values of the workplace culture aligned with those of NQNs, and they act accordingly, they experience a sense of belonging and the feeling of meaningful work is maintained. This alignment strengthens their self-esteem and overall well-being [22]. The sense of meaningfulness seems to be linked with increased job satisfaction and retention [42, 44]. Collegial support has been shown to enhance confidence and self-efficacy [10, 18]. The quantitative descriptive results of this study show that the meaning of work, social support from colleagues, and sense of community received the highest ratings over time, as also reported in recent studies [5, 7, 9, 11].

NQNs in this study reported that a heavy workload and a fast-paced workplace culture may lead to increased stress, which can hinder the development process and result in lower self-confidence. However, a workplace culture characterised by collaboration and fellowship was found to help NQNs prioritise effectively and cope with hectic situations. NQNs who worked in workplaces characterised by an unsupportive culture, where they encountered colleagues who were not conducive to their need to learn and develop, could lead to feelings of abandonment, alienation, and ill-being, as also mentioned in earlier studies [4, 7, 9, 11].

The descriptive data are consistent with and confirm the findings in the qualitative data, thus strengthening the evidence of the importance of collegial support and a caring workplace culture. This is consistent with a previous study, which concluded that supportive workplace cultures can compensate for insufficient mentorship during the transition [3]. Moreover, this study emphasises the importance of a value-based workplace culture and collegial support in facilitating the development of NQNs into confident nurses, thereby ensuring high-quality patient care and nurse retention.

### Methodological strengths and limitations

A multimethod (QUAL-quan) triangulation design was selected to evaluate the conditions necessary to realise the mechanisms of change of an intervention over seven months [26, 45]. Since this post-intervention evaluation aimed for a deeper understanding, it was driven by a qualitative research approach, wherein the quantitative study served to facilitate the interpretation of the qualitative findings and identify potential changes over time. The small sample size (*n* = 27) is a limitation, given that only 56 % of the participants completed the survey. The follow-up study did not show any change over time, so it is not possible to demonstrate any effects. A significant threat to the study was the ongoing COVID-19 pandemic, which had a substantial impact on the study. No physical meetings, workshops or contacts were allowed, meaning that all activities and interviews were conducted online. This may be one reason for the high drop-out rate, in addition to the high workload, sick leaves, and change of workplace.

A strength of the study is that the focus group interviews provided rich and nuanced data, and by using the same interview guide in both countries, the trustworthiness of the interviews was strengthened. In summary, the advantages of using a multi method triangulation design in evaluating this intervention among NQNs outweigh the limitations of this study. The findings of this study can be transferred with caution to other healthcare contexts.

## Conclusion

The findings of this study demonstrate the considerable importance of developing a caring workplace culture that welcomes and respects NQNs as new colleagues. Value-conscious leadership is pivotal in fostering such a culture by promoting the articulation and setting of the tone in the workplace, thereby creating conditions that support continuous learning, fellowship, and overall well-being. Therefore, it is essential to focus on NQNs’ workload and capacity by fostering a supportive workplace culture, ensuring appropriate workloads, providing comprehensive induction, and clearly defining roles. By fostering collaborative, peer, and collegial learning NQNs’ personal and professional development is supported. This study contributes to strengthening the evidence of the importance of a caring workplace culture and supportive colleagues in fostering confidence among nurses. Further research should focus on international interventions and incorporate follow-up studies with larger samples to assess the impact of workplace culture and psychosocial factors on staff retention and the quality of patient care. Further investigation is necessary to understand how different leadership support strategies might influence NQNs’ well-being and their motivation to stay in the profession.

## Supplementary Information

Below is the link to the electronic supplementary material.

Supplementary Material 1

## Data Availability

The datasets generated and/or analysed during the current study are not publicly available due to privacy or ethical restrictions, but are available from the corresponding author on reasonable request.

## Abbreviations

COPSOQ III: Copenhagen Psychosocial Questionnaire III
COREQ: Consolidated criteria for reporting qualitative research
GRAMMS: Good reporting of a mixed methods study
OSE: Occupational self-efficacy
NQN: Newly qualified nurse
