# Supplementary material for: The importance of collegial support and a caring workplace culture for newly qualified nurses in becoming confident during their transition: a multi method study
Source: BMC Nurs. 2025 Nov 22;24:1502. doi: 10.1186/s12912-025-04137-y (PMC12752334; doi:10.1186/s12912-025-04137-y)
Supplement: Supplementary file 1 — Supplementary Material 1 [file 12912_2025_4137_MOESM1_ESM.docx]

# Interview Guide

## Focus Group Interviews with Mentees Participating in the Intervention

During the Focus Group interview, we will discuss a) the support you have received during the intervention, 09/2021–04/2022, and its significance for your development as a nurse, b) your development as nurses during this period, and c) what good care entails.

## Can you please tell us about:

1. The importance of the workplace culture for your development as a nurse
2. What, if anything, within the workplace culture has supported your growth?
3. What, if anything, within the workplace culture has hindered your development?
4. The support you received for your development as a nurse
5. What was especially important or meaningful for your development?
6. Is there any support you feel you have lacked? If so, what?
7. What does good care mean to you as a nurse?
8. What core values inspire you in your work?
9. What are your thoughts on your future development and career?
